# Supplementary material for: Evolution of Thyroglobulin Loop Kinetics in EpCAM
Source: Life (Basel). 2021 Sep 3;11(9):915. doi: 10.3390/life11090915 (PMC8467770; doi:10.3390/life11090915)
Supplement: Supplementary file 1 [file life-11-00915-s001.zip › life-1362516-supplementary.pdf]

Supplementary Information

Evolution of Thyroglobulin Loop Kinetics in EpCAM

Serena H. Chen[a*] and David R. Bell[b*]

[a] Computational Sciences and Engineering Division, Oak Ridge National Laboratory, Oak Ridge, TN 37830, USA

[b] Advanced Biomedical Computational Science, Frederick National Laboratory for Cancer Research, Frederick, MD 21701, USA

* To whom correspondence should be addressed. E-mail: chens@ornl.gov (S.H.C.), david.bell@nih.gov (D.R.B)

Notice: This manuscript has been authored by UT-Battelle, LLC under Contract No. DE-AC05-00OR22725 with the U.S. Department of Energy. The United States Government retains and the publisher, by accepting the article for publication, acknowledges that the United States Government retains a non-exclusive, paid-up, irrevocable, worldwide license to publish or reproduce the published form of this manuscript, or allow others to do so, for United States Government purposes. The Department of Energy will provide public access to these results of federally sponsored research in accordance with the DOE Public Access Plan (http://energy.gov/downloads/doe-public-access- plan).

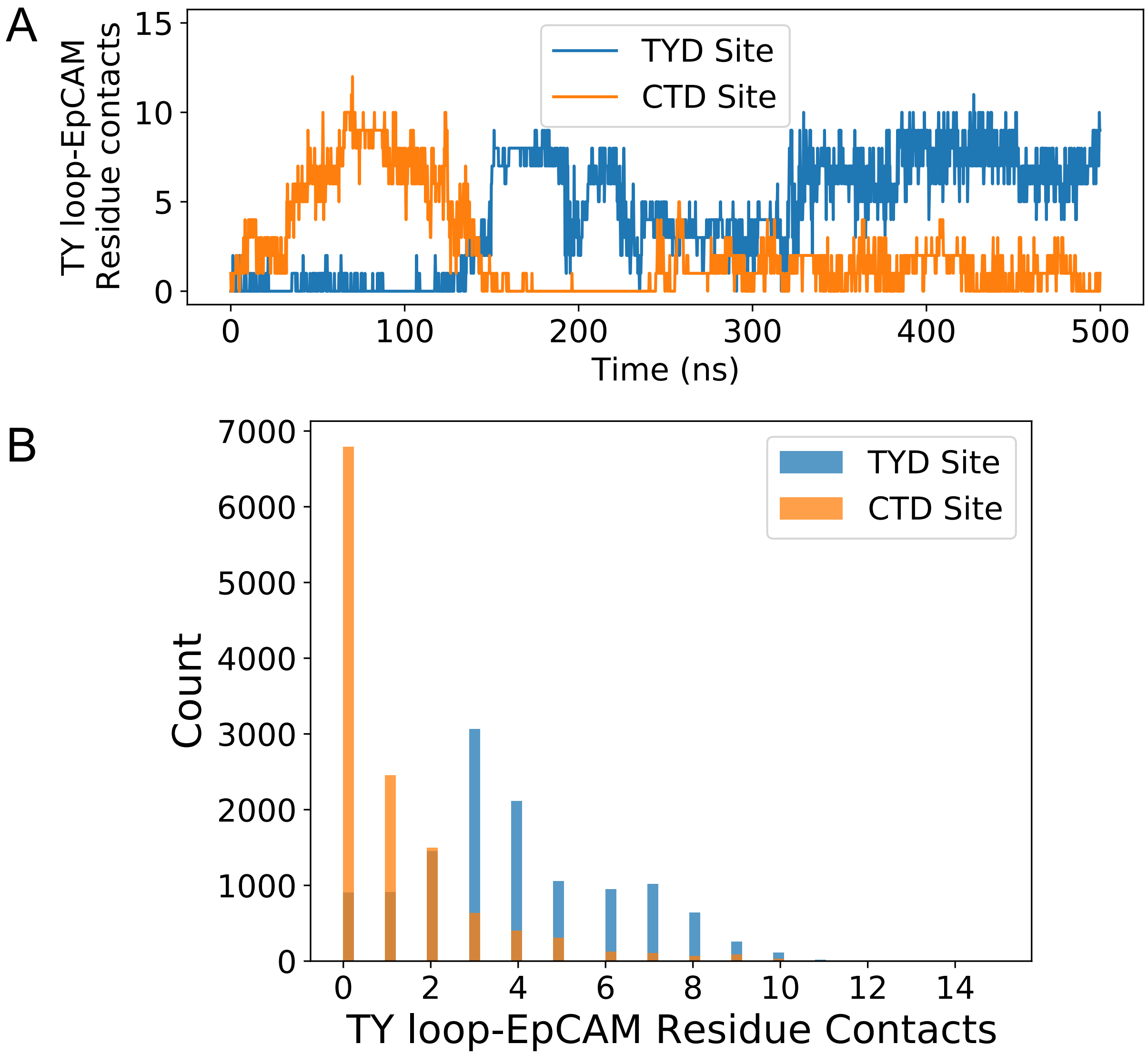

Figure S1. Human TY loop-EpCAM residue contacts. (A) TY loop-EpCAM residue contacts over time of a representative trajectory. (B) Histogram of TY loop-EpCAM residue contacts of all trajectories. Residue contacts are defined as the number of TY loop residues contacting at least one residue of the respective EpCAM site. A residue pair is in contact if any atoms are within 4.5 Å. There is an exponential decay for the TY loop-CTD site residue contact counts. However, for the TYD site, there is a clear peak at three residue contacts, i.e., three of the TY loop residues contact at least one residue of the TYD site. We thus used three residue contacts as criteria for defining TYD_{closed} and CTD_{closed} forms.

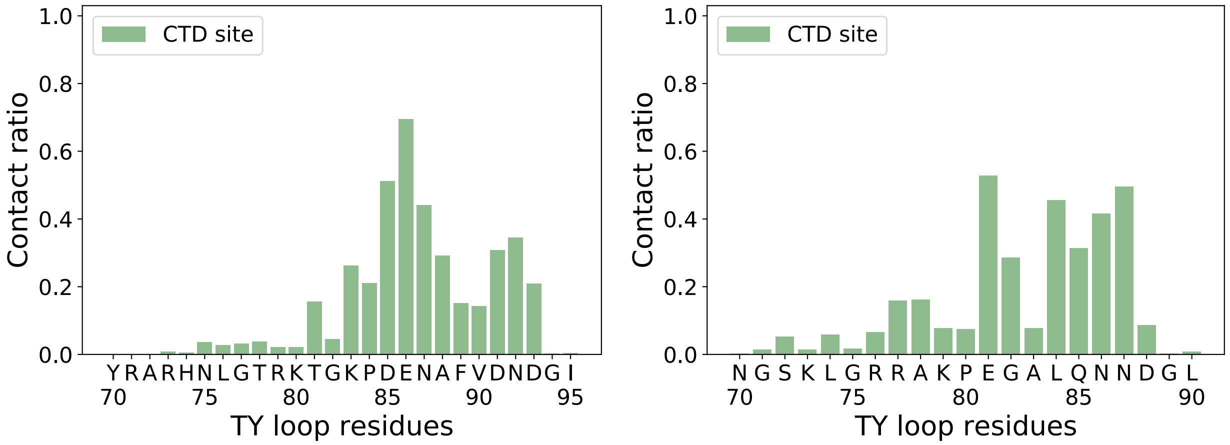

Figure S2. Contact ratio of TY loop residues in the CTD_{closed} form for Fish (left) and Fish+HTY (right) systems. The contact ratio for the CTD site is defined as the number of structures that a TY loop residue is in contact with the CTD site to the total number of structures in the CTD_{closed} form. A residue pair is in contact if any atoms are within 4.5 Å.
